# Helical Structures Mimicking Chiral Seedpod Opening and Tendril Coiling

**DOI:** 10.3390/s18092973

**Published:** 2018-09-06

**Authors:** Guangchao Wan, Congran Jin, Ian Trase, Shan Zhao, Zi Chen

**Affiliations:** Thayer School of Engineering, Dartmouth College, Hanover, NH 03755, USA; guangchao.wan.th@dartmouth.edu (G.W.); congran.jin.th@dartmouth.edu (C.J.); ian.trase.th@dartmouth.edu (I.T.); shan.zhao@dartmouth.edu (S.Z.)

**Keywords:** biomimetic, seedpod opening, tendril coiling, helical structures, stimuli-responsive materials, perversion

## Abstract

Helical structures are ubiquitous in natural and engineered systems across multiple length scales. Examples include DNA molecules, plants’ tendrils, sea snails’ shells, and spiral nanoribbons. Although this symmetry-breaking shape has shown excellent performance in elastic springs or propulsion generation in a low-Reynolds-number environment, a general principle to produce a helical structure with programmable geometry regardless of length scales is still in demand. In recent years, inspired by the chiral opening of *Bauhinia variegata*’s seedpod and the coiling of plant’s tendril, researchers have made significant breakthroughs in synthesizing state-of-the-art 3D helical structures through creating intrinsic curvatures in 2D rod-like or ribbon-like precursors. The intrinsic curvature results from the differential response to a variety of external stimuli of functional materials, such as hydrogels, liquid crystal elastomers, and shape memory polymers. In this review, we give a brief overview of the shape transformation mechanisms of these two plant’s structures and then review recent progress in the fabrication of biomimetic helical structures that are categorized by the stimuli-responsive materials involved. By providing this survey on important recent advances along with our perspectives, we hope to solicit new inspirations and insights on the development and fabrication of helical structures, as well as the future development of interdisciplinary research at the interface of physics, engineering, and biology.

## 1. Introduction

Helical structures behave as building blocks in Nature across several length scales ranging from nanoscale DNA macromolecules and bacterial flagella [1] to millimeter-scale plant tendrils [2] and sea snail shells [3]. The helical shape lacks mirror symmetry and plays an essential role in many biological processes such as seeds’ penetration in soil [4], the motion of *Escherichia coli (E. coli)* in body fluids [5], and in various engineering applications, including the propulsion of a swimming micro-robot in low-Reynolds-number [6] or granular environments [7] and in stretchable electronic devices [8]. Although excellent works have demonstrated the accessibility of fabricating helical structures ranging from nanoscale [9] to macroscale [10], the direct fabrication of a 3D helix still poses a great challenge, especially at small sizes. One feasible solution is to rely on the shape transformation from a 2D configuration to a 3D helical shape, and this topic has attracted intense attention from applied mathematicians, physicists, and engineers alike. The motivation of this subject comes from the fact that many developed and sophisticated techniques such as photolithography, layer-by-layer deposition and electrospinning can facilitate the synthesis of a 2D ribbon-like or rod-like structures with complex yet tunable internal constitutions.

Nature teems with excellent examples of this type of shape transition. One example is the chiral opening process of *Bauhinia variegata*’s pods [11], where an initially flat pod valve changes its shape into a 3D helix via a hygroscopic process. Another example is the coiling of a plant’s tendrils such as *Towel Gourd* [12] and cucumber (*Cucumis sativus* or *Echinocystis lobata*) [13]. Initially, the soft tendril circumnutates itself and its tip follows a looping trajectory [14]. Once the tendril touches a support, it enters a new growing period and becomes woody and strong. During this stage, the tendril further coils itself and forms a perversion that connects two helical sections with opposite chirality, and this spring-like structure can contribute to resisting strong wind and other biological advantages.

For the seedpod opening, the underlying mechanism originates from the oriented cellulose fiber’s distribution inside the matrix [11]. The fiber reinforcement restricts the swelling/deswelling deformation of the organic matrix along the fiber’s direction without much impact on the deformation perpendicular to the reinforcement direction when exposed to the moisture change in environment. As a result, anisotropic deformation of the plant’s matrix generates an intrinsic saddle configuration that can only be accommodated by transforming the planar shape into a 3D helical configuration. For the tendril’s coiling, a hierarchically chiral structure that spans several length scales allows the transfer of individual cell chirality into macroscopic tendril coiling for the initial growing stage before the tendril attaches to a support. Once in contact with a support, the dominating coiling mechanism changes. The asymmetric contraction of cells inside the tendril forms an intrinsic curvature due to differential lignification [13]. The produced intrinsic curvature, along with topological constraints since the tendril’s ends cannot freely rotate, leads to a helical tendril with dual chirality—each half portion of the tendril is a helix with opposite chirality. There is a small section linking these two helices, often referred as a ‘perversion’.

The shape-shifting mechanism of pod’s opening and tendril’s coiling is quite generic and puts no restriction on the length scale of the target shape—that is, to create an intrinsic curvature inside a 2D straight rod or flat ribbon. Here, the term ‘intrinsic’ represents the stress-free shape or the shape containing the minimum elastic energy [15]. Differential growth or contraction of internal cells during environmental variation or biological growth is utilized by these two plants to produce the intrinsic non-planar configuration, and this can be replicated in artificial systems by using the differential response of stimuli-responsive materials. The famous pioneering work with respect to this subject is probably the bending of a bi-metal beam from Timoshenko [16].

Here we provide a brief review on recent progress in the synthesis of helical structures relying on shape transition from a 2D rod-like or ribbon-like configuration to a 3D helix mimicking *Bauhinia variegata*’s pod opening and tendril coiling. Our focus is to illustrate the shape-shifting mechanisms of these two plants and introduce stimuli-responsive materials that can be tailored to achieve this goal. In Section 2, helical structures inspired from *Bauhinia variegata* are introduced according to the type of functional materials used, including hydrogels, liquid crystal networks/elastomers, and shape memory polymers. Each subsection is further organized based on the kind of responsive stimuli, such as heat or light. In Section 3, we concentrate on the coiling mechanism of tendrils, specifically *Towel Gourd* and cucumber tendrils, and present some biomimetic examples that exhibit the similar behavior. Carbon-nanotube-based helical fibers that consist of hierarchical chiral building blocks are introduced as examples following the coiling mechanism before the tendril finds the support. Then artificial helical structures with one or multiple perversions inspired from the tendril’s coiling after it attaches to a support are presented. These can arise in a bilayer elastomeric strip or an electrospun fiber with a heterogeneous structure. Finally, we close with an outlook on the potential opportunities and future challenges in the field.

## 2. Helical Structures Mimicking the Opening of *Bauhinia Variegata* Pods

### 2.1. Opening Mechanism of Bauhinia Variegata Pods

#### 2.1.1. Hygroscopic Motion in Plants

Plants can move in response to external stimuli such as a change in humidity or the approach of prey. Examples include the snapping of the Venus flytrap (*Dionaea muscipla*) [17], the opening of pine cones [18], and the seed dispersal of wheat awns [19]. Different from muscle-actuated animal motion, plant motion results from water-driven swelling or shrinkage of cells inside the tissue [20]. During these hygroscopic processes, the swelling or shrinkage of the tissue will usually be guided by the microstructure of the cell wall where aligned cellulose fibrils are embedded. Resembling artificial fiber-reinforced composites, tissue deformation along the fiber’s alignment will be restricted while shape change perpendicular to the fiber’s orientation will be less affected. This anisotropic deformation contributes to the sophisticated morphogenesis of plants.

**Figure 1:**
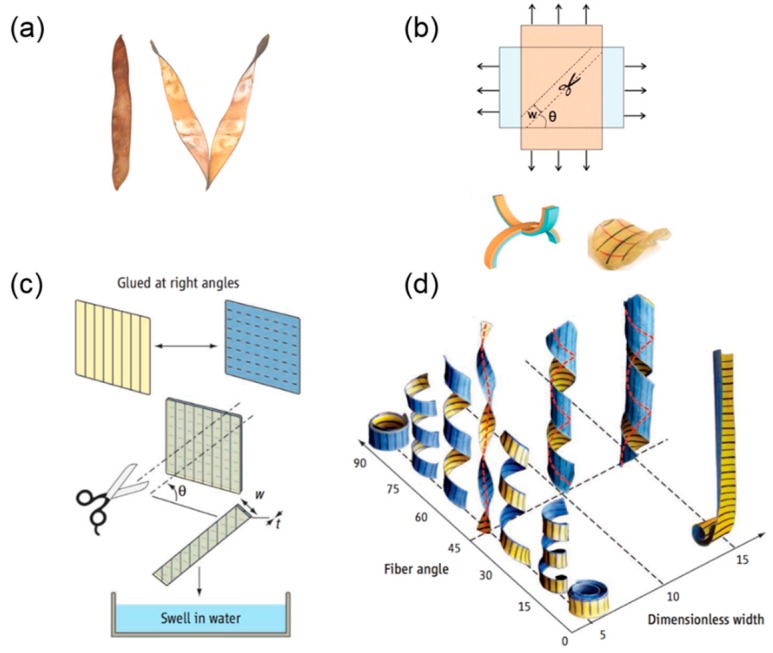
The opening mechanism of *Bauhinia variegate*’s pod. (**a**) A closed seedpod in a wet environment (left) and the opened seedpod in a dry environment (right); (**b**) Mechanical analog of seedpod opening by attaching two uniaxially stretched elastomer sheets together and cutting a ribbon at an angle θ with width *w* (top). The bilayer sheet adopts a saddle shape: schematic illustration (bottom left), experiment (bottom right); (**c**) The process to generate helices using a bilayer paper sheet reinforced with fibers; (**d**) Design space for helix generation with respect to the fiber angle and the dimensionless width. (**a**,**b**) are from [11], reprinted with permission from AAAS; (**c**,**d**) are from [22], reprinted with permission from AAAS.

One well-known example of a humidity-driven plant shape transformation is the opening process of *Bauhinia variegata*’s pod valves (Figure 1a), where a flat valve twists itself into a helix. The underlying mechanism of this seedpod’s opening is illustrated in [11]. The microstructure of this seedpod consists of two fibrous layers. The fibers’ orientation in one layer is perpendicular to the other, and both have a 45° angle relative to the long axis of the seedpod. When the air is dry, the cells in the sclerenchyma tissue will lose water and make the tissue matrix shrink. Owing to the cellulose fiber reinforcement, shrinkage only occurs perpendicularly to the fiber’s orientation in each layer, and thus two layers in the seedpod valve shrink perpendicularly to each other. As a result, the thin pod’s valve prefers to adopt a saddle shape by bending into opposite curvatures along two orthogonal directions. 

The emergence of this intrinsic saddle shape can be proven by the mechanical analog in which two uniaxially stretched elastomer sheets are attached together. If we cut a small piece from this bilayer elastomer sheet, it will assume a saddle shape at rest (Figure 1b). This intrinsic saddle shape will drive the flat pod into a helix, which can be predicted using the non-Euclidean theory [21].

#### 2.1.2. Transition from Pure Twisting to Helical Coiling During Pod Opening

A helical curve can be uniquely determined by two parameters, the radius *r* and the pitch *p* (the pitch’s sign determines the handedness of a helix). Experiment and theoretical analysis show that the radius and pitch of the final helical seedpod are determined by several parameters including the intrinsic curvature $\kappa_{0}$, the angle between the fiber’s orientation and seedpod’s long axis θ, the seedpod thickness *t*, and the width *w*. The influence of these multiple parameters can be generalized into a dimensionless width $\tilde{w}=w\sqrt{\kappa_{0}/t}$, which reflects the relative magnitude of the stretching energy and bending energy [11,23]. Forterre et al. [22] provided an overview of the parametric study with respect to this dimensionless width $\tilde{w}$ and the fiber misoriented angle θ (Figure 1c). They mimicked the pod’s microstructure by aligning fibers inside a paper sheet and bonding two sheet together. The fiber’s direction in one sheet is orthogonal to the other and both have a tilting angle with respect to the longitudinal axis of the cut ribbon. A helix can be generated when the bilayer is swollen in water. 

When $\tilde{w}\gg 1$, stretching is dominant. The Gaussian curvature $K=\kappa_{1}\kappa_{2}$ ($\kappa_{1}$ and $\kappa_{2}$ are the two principal curvatures) is zero everywhere to avoid planar stretching except at a boundary layer with a characteristic length scale $\sqrt{t/\kappa_{0}}$ [11,23,24]. The seedpod coils into a cylindrical helix along either of the two principal directions of the intrinsic saddle shape. When $\tilde{w}\ll 1$, the energy cost from bending is high while stretching is favorable. The Gaussian curvature K can be negative while the mean curvature $H=\left(\kappa_{1}+\kappa_{2}\right)/2$ will be zero to minimize bending energy. As a result, the seedpod twists itself while keeping the centerline straight (helicoid). A transition from pure twisting to helical coiling as $\tilde{w}$ increases is not only observed in the pod valve, but can be also demonstrated via the aforementioned mechanical analog [25]. Figure 1d quantitatively shows the impact of the fiber angle θ and dimensionless width $\tilde{w}$ on the helix shape. When θ=45° and $\tilde{w}$ is small, the ribbon twists itself into a helicoid with a straight centerline. Deviation from this scenario will increase radius *r* and decrease pitch *p* accordingly, forming a spiral helical shape until it rolls up into a circular shape when θ=90° or 0°.

### 2.2. Biomimetic Helical Structures Based on Stimuli-Responsive Materials

The mechanism of chiral pod opening shows that a helix can be generated from a 2D ribbon-like structure if an intrinsic saddle configuration is created and its principal direction has a tilting angle with respect to the long axis of the ribbon. This mechanism is universal and not limited by the length scale of the entire structure, which provides a meaningful pathway to producing helical shapes across different length scales from a 2D precursor. Inspired from differential swelling or deswelling of cells inside the pod, it is straightforward to employ the differential response of stimuli-responsive materials that are combined into one single structure to generate intrinsic configuration when subjected to the external stimuli. Sophisticated techniques such as layer-by-layer deposition and photolithography can accomplish this by patterning 2D structures with through-thickness or planar composition variation. Based on this idea, numerous stimuli-responsive materials have been tailored such as hydrogels, liquid crystal networks and elastomers, and shape memory polymers. Some recent works utilizing these three types of stimuli-responsive materials to fabricate helical structures are listed in Table 1 and organized in terms of their corresponding stimuli, length scale, and actuation time. 

In addition to the actuation of stimuli-responsive materials, there are also many methods that can fulfill a similar purpose. One can combine two layers with a uniaxial pre-stretch in perpendicular directions [45]. It is also verified that using chiral molecules and building blocks in a single layer is mathematically equivalent to the formation of an intrinsic saddle shape [28]. Such universality makes *Bauhinia variegata*’s pods an outstanding prototype to inspire further fabrication of helical structures.

Here, we focus on the helical structures made of stimuli-responsive materials. The following sub-sections are organized based on the type of the functional material used, among which are hydrogels, liquid crystal networks/elastomers, and shape memory polymers. We hope that this summary provides useful information for researchers from different fields who are interested in designing and fabricating innovative biomimetic helical structures.

#### 2.2.1. Hydrogel-Based Helical Structures

Hydrogels include a broad range of polymers and can be roughly defined as three-dimensional networks swollen by a solvent [46]. Compared to other stimuli-responsive materials, hydrogels can achieve reversible volume changes up to several folds when subjected to multiple stimuli such as humidity, temperature, ionic strength, or light. They provide an efficient pathway to realizing large actuation via different controls. Besides, the open networks of hydrogels allow for subsequent chemical modification such as the formation of interpenetrating networks and for embedded additives such as stiff reinforcement or functional nanoparticles. This endows a variety of options to design actuators that can respond to different types of external signals. For further information, one can refer to Ref. [47].

Bulk hydrogels can only swell or deswell isotropically, resulting in simple in-plane elongation and contraction. More complex shape transformation relies on the hybrid hydrogels in which the material composition varies either through the thickness [48] or along the planar directions [49,50,51]. Hu et al. [52] first reported the utilization of hydrogel to fabricate a bilayer beam that bends in response to mismatched strain between two layers. This pioneering work has inspired interest in hydrogel-based shape morphing structures. Recently, a similar idea was used to construct smart hinges that can fold or unfold microscale origami [53]. Utilizing an interpenetrating network formed through photo-crosslinking, Wu and co-workers [54] successfully fabricated a single-layer heterogeneous sheet with in-plane composition variation. The interaction between adjacent regions generates internal stress that buckles the planer sheet into various three-dimensional configurations such as helices, domes, or saddle shapes [55]. In their works, the authors harnessed material heterogeneity to enable a single sheet to respond to different stimuli, since each individual composition is sensitive to a specific stimulus. This greatly broadens the number of actuation modes available to a single hydrogel sheet [55,56].

In this sub-section, we describe hydrogel-based helical structures that mimic the chiral opening of *Bauhinia variegata’s* pod sorted by the type of stimulus: humidity, temperature, and pH. Inspired by the pod’s microstructure, in which the cellulose fibrils are aligned in cells, researchers embedded stiff reinforcement in hydrogels to guide the anisotropic swelling or deswelling of the hydrogels once actuated. In addition, differential response through the thickness direction is necessary so that the intrinsic curvature will accommodate the mismatch strain [57], leading to the shape transformation from a flat ribbon to a helix.

##### Humidity-Responsive Hydrogels

Water molecules can diffuse into or out of a hydrogel when humidity changes. When water is adsorbed, the space between polymer chains increases and the hydrogel swells. This shape change is isotropic, and its speed mainly depends on the rate of water diffusion. 

Learning from nature, one could use fiber-like reinforcements to restrict deformation along the reinforced direction [58]. The isotropic swelling or deswelling of a hydrogel can thus be tailored to be anisotropic [59]. For instance, Zhang et al. [60] buried glass fibers inside agarose hydrogel as a reinforcement to guide the bending of a hydrogel ribbon when it is exposed to humidity on one side. 

**Figure 2:**
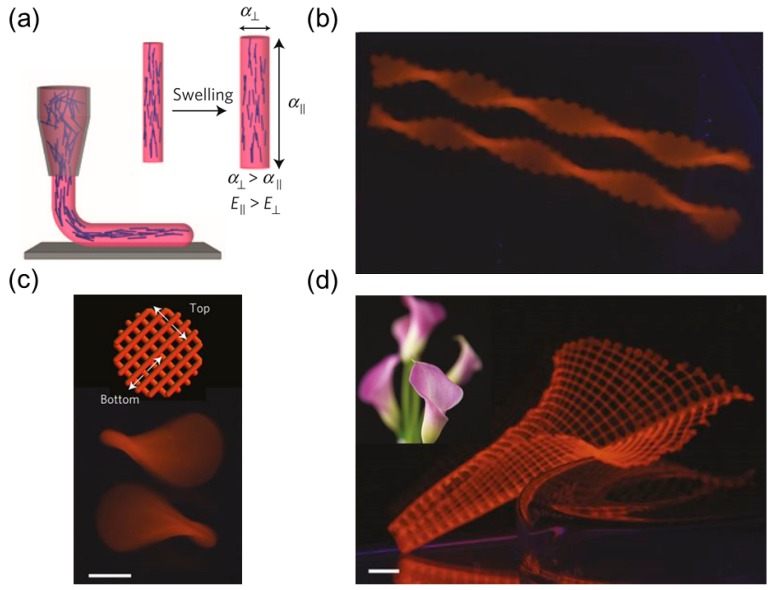
Humidity-responsive hydrogel-based helical structures mimicking *Bauhinia variegata*. (**a**) Shear-induced alignment of cellulose fibrils along the printed filament (left) and anisotropic swelling of the printed filament (right); (**b**) Helical shape generated by 4D-printed hydrogel; (**c**) Saddle shape (bottom) from orthogonal patterns of filaments in two layers (top), the scale bar is 2.5 mm; (**d**) Biomimetic lily flower from 4D-printed hydrogel, the inset is the lily flower in nature and the scale bar is 5 mm. (**a**–**d**) are from Ref. [26], reproduced with permission, copyright 2016 Nature Publishing Group.

Gladman et al. [26] mimicked the seedpod microstructure by 3D-printing the cellulose fibrils inside the hydrogel matrix. By harnessing shear-induced alignment when the ink is ejected from the nozzle, the cellulose fibrils are aligned along the longitudinal direction of the printed filament and hence it is possible to locally control the fibril direction (Figure 2a). 

Once the hybrid hydrogel is swollen in solution, the transverse swelling ratio of the filament becomes larger than the longitudinal swelling ratio (Figure 2a). By printing two layers of parallel filaments and keeping the filament’s direction in one layer perpendicular to the other, the intrinsic saddle shape can be constructed based on the filament’s anisotropic swelling (Figure 2c). If the filaments are aligned at ±45° relative to the long axis of the hydrogel ribbon, it will yield a twisted helix (Figure 2b).

The 3D-printing of stimuli-responsive materials that can change shape with time in a programmable way is sometimes referred to as 4D printing. This technique gives one more tunable variable to the 3D printing, which makes it a powerful tool for designing stimuli-responsive structures [61].

The advantage of this experimental prototype is that the 3D printing allows the filaments to be arranged horizontally in plane or vertically layer-by-layer based on the printing path. In-plane filament’s pattern allows for tunable stretching while vertical filament’s arrangement allows for bending. Accordingly, the Gaussian curvature *K*, which is related to the local stretching, and the mean curvature *H*, which depends on the bending, can be separately controlled. This offers the possibility to design any arbitrary surface on demand, such as a calla lily flower (Figure 2d).

**Figure 3:**
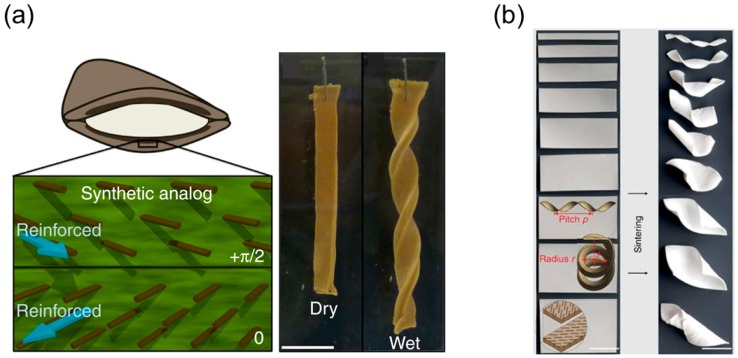
Humidity-responsive hydrogel-based helical structures mimicking *Bauhinia variegata*, the reinforcement’s distribution is controlled via magnetic field. (**a**) Microplatelet alignment in a bilayer sheet (left, schematic) mimicking *Bauhinia variegata* and shape transformation of the synthesized hydrogel in water (right). The scale bar is 1 cm. (**b**) Microplatelet-induced shape transformation of ceramics from a planer sheet (left) to a helical shape (right) after sintering, the radius and pitch of the helix are influenced by the ribbon’s width from top to bottom (right). The scale bar is 25 mm; (**a**) is from [27], reproduced with permission; (**b**) is from [65], reproduced with permission, copyright 2013 Nature Publishing Group.

Apart from a direct 3D printing, the direction and distribution of the reinforcement can also be remotely controlled using magnetic [62,63] or electric fields [64]. Erb and collaborators [27] used ultra-low magnetic fields to guide the orientation of microplatelets (Al_2_O_3_) coated with superparamagnetic nanoparticles in a precursor solution. The orientation of the platelets was preserved during polymerization. By using two-step layer-by-layer fabrication and changing the magnetic field direction in each step, they obtained a bilayer hydrogel ribbon whose microstructure mimics *Bauhinia variegata’s* pod. Like the seedpod, this hybrid hydrogel ribbon can either purely twist itself as a helicoid or coil into a spiral helix (Figure 3a) depending on the ribbon’s width and reinforcement orientation. This fabrication method puts no strict restriction on the matrix as the precursor solution does not severely retard the alignment of microplatelets during the curing process, which substantially broadens the scope of available materials and responsive stimuli. 

For example, such a mechanism can be realized via humidity-responsive gelatin, thermal-responsive hydrogel (poly(*N*-isopropylacrylamide) (PNIPAm) or even rigid ceramics [65] that shrink during the sintering process, and a similar transition from a helicoid to a spiral helix is also observed when the width increases in these ceramics (Figure 3b). This prototype can locally control the microplatelet orientation on demand, enabling programming the target shape by spatially varying the reinforcement directions.

##### Thermally Responsive Hydrogels

Some hydrogels will shrink when the temperature exceeds a low critical solution temperature (LCST) [46]. One example is PNIPAm, commonly used as a thermally responsive material for actuation purposes. When the temperature reaches the LCST, a hydrophobic interaction between polymer chains leads to a molecular transition from hydrophilic coil to hydrophobic globule [63], expelling water from the network.

Thermally responsive hydrogels can be actuated by either directly increasing the temperature of the surrounding environment or by incorporating heat generating additives inside the network. Yu and coworkers harnessed the Joule heat generated by the embedded electric circuits to control the swelling and deswelling of bulk hydrogel PNIPAm [66]. Hayward’s group utilized the surface plasmon resonance of gold nanoparticles to convert light at specific wavelengths into thermal energy to bend [67] or buckle a gel sheet [68]. The responsive wavelength is controlled by the size of the nanoparticles. The near-infrared photothermal property of carbon-based materials such as carbon nanotubes [69] or reduced graphene oxide [70,71] can also be employed to achieve fast, reversible actuation of thermally responsive hydrogels.

**Figure 4:**
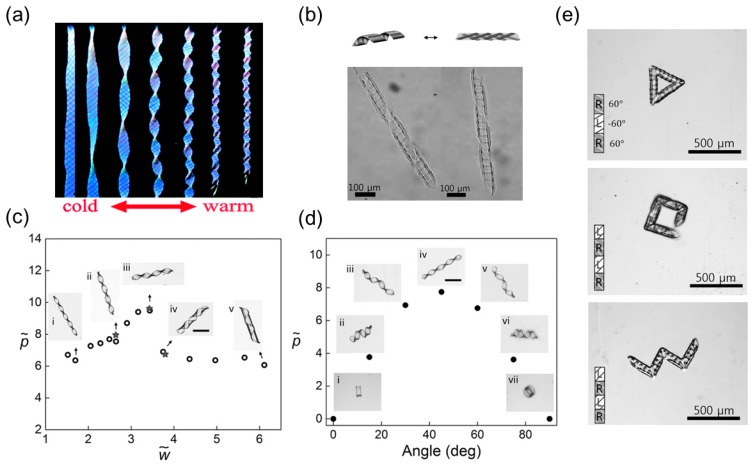
Thermally responsive hydrogel-based helical structures mimicking *Bauhinia variegata*. (**a**) Transition from a twisted helicoid to a spiral helical shape as the shrinkage ratio of hydrogel increases with rising temperature; (**b**) Schematic illustration of helical transformation in a tri-layer hydrogel composite (top) and a helical hybrid hydrogel with right-handedness (bottom left) or left-handedness (bottom right) under an optical microscope; (**c**) The relationship between the helix pitch and the dimensionless width; (**d**) The relationship between the helix pitch and the reinforcement angle; (**e**) Complex shapes obtained by connecting helices with different lengths or handedness, including triangle (top), square (middle) and zigzag (bottom). The insets are 2D precursors of the hydrogel sheets during photo-crosslinking. (**a**) is from [28], reprinted with permission from The Royal Society of Chemistry; (**b**–**e**) are from [29], reprinted with permission from 2017 Wiley.

The response of thermal-responsive hydrogels, like the humidity-responsive hydrogels, is isotropic (invariant with respect to directions). Therefore, additive reinforcement is necessary to guide the swelling and deswelling direction. Armon et al. [28] embedded rigid threads in the top and bottom surfaces of a NIPA gel sheet as fiber reinforcements. When the temperature rises beyond the LCST, the NIPA gel will shrink and twist itself into a helicoid or a helix from the thread’s restriction (Figure 4a). The transition from pure twisting to spiral helix happens as the shrinking ratio increases, in agreement with the theoretical analysis in which the dimensionless width $\tilde{w}$ is related to the swelling/shrinkage induced curvature $\kappa_{0}$ as $\tilde{w}=w\sqrt{\kappa_{0}/t}$.

Jeon and coworkers [29] photo-crosslinked a microscale tri-layer structure. The middle layer is the soft hydrogel poly(*N*,*N*-diethylacrylamide-*co*-acrylamidobenzophenone) (PDEAM-BP) and the outer layers are parallel strips made of rigid, passive polymers. The orientation of strips in one outer layer is kept orthogonal to those in the other outer layer. The strip pattern, including the angle relative to the long axis of the ribbon, the width, and the spacing is controlled by the photomask design. Upon swelling in a buffer solution, the flat ribbon will coil into a helix (Figure 4b), the handedness, pitch and radius of which are determined by the dimensionless width $\tilde{w}$ and the angle of the parallel strips (Figure 4c–d). This relationship resembles the previous theoretical analysis performed by Armon et al. [11]. A sharp decrease in pitch is found when the dimensionless width exceeds the critical point and thus a twisted shape will transition into a spiral helix (Figure 4c). Besides, a 45° tilting angle will produce a twisted helix, and 0° or 90° angle will give a ring shape (Figure 4d). Since the hydrogel is also responsive to heat, increasing temperature will decrease the swelling ratio of the middle layer and untwist the helix. 

Here, the patterned, rigid polymer strips in Jeon’s work play an equivalent role as the embedded fiber reinforcement in offering preferential swelling and deswelling direction to the middle, soft layer, and the orthogonal strip alignments in the top and bottom layers form an intrinsic saddle shape that drives the flat ribbon into a helical shape. Optical microscopy images show that the strips on the outer side of the helix orient along the helix axis (Figure 4b) to minimize the stretching and bending in these strips [72]. 

One advantage of photo-crosslinking is to create an arbitrary 2D geometric pattern with high spatial resolution. More complex shapes at the microscale can be acquired by patterning strips with different angles on specific regions of one monolithic hydrogel ribbon, yielding concatenated helices. Various shapes such as a zigzag, square, or triangle can be produced by tuning the strip angles in different regions (Figure 4e). Jeon et al. also demonstrated that the block angle, torsion angle, and length of the connected helices can be separately programmed, providing a feasible method to fabricate arbitrary 3D curves in microscale. 

##### pH Responsive Hydrogels

Polymer chains in hydrogels can carry ionic groups, and the network usually stays neutral since the oppositely charged ions balance themselves [46]. However, an environment change such as pH variation will cause the ions to diffuse into or out of the network, resulting in negatively or positively charged chains. The electrostatic repulsion between the charged chains or the destruction of the previous physical crosslinks such as hydrogen bonds increases the interspace, and the hydrogel swells. 

**Figure 5:**
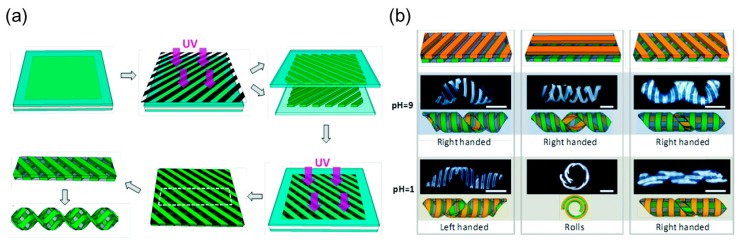
pH-responsive hydrogel-based helical structures mimicking *Bauhinia variegata*. (**a**) Schematic illustration of the three-step photo-crosslinking of PAA/PNIPAm hybrid composite guided by a photomask. The green strips are PAA while the grey part is PNIPAm. The photomask is made by drawing black lines; (**b**) Shape transformation of P(VI-*co*-AAM)-PNIPAm-PAA hybrid hydrogel when pH changes from 9 to 1. The brown, grey and green parts are P(VI-*co*-AAM), PNIPAm and PAA, respectively. The upper array shows the strip orientations in the top and bottom layers. The schematic (bottom) and experimental (top) figures of shape transformation are both shown together in the middle and bottom array. The scale bar is 1 cm (**a**,**b**) are from [30], reprinted with permission from The Royal Society of Chemistry.

Based on the synthesis of an interpenetrating network by photo-crosslinking the monomer solution of one gel in another formed hydrogel matrix, Wang and coworkers [30] used a three-step photopolymerization to fabricate a hybrid hydrogel. They first photo-crosslinked parallel strips of PAA (poly (acrylic acid)) guided by a photomask and left the strips on the templates (Figure 5a top middle). Then they placed two templates face to face with a tilting angle between the strip orientations in each template (Figure 5a, top right). The gap between two templates is controlled using spacers. Injecting a solution of PNIPAm inside the space between the two templates and photo-crosslinking the PNIPAm precursor will form a heterogenous structure that contains an interpenetrating network of PAA/PNIPAm and a single network of PNIPAm (Figure 5a, bottom right). The parallel PAA/PNIPAm strips act like cellulose fibers while the single network PNIPAM resembles the organic matrix, mimicking the microstructure of *Bauhinia variegata*’s pod. Since PAA/PNIPAm swells in basic solution while PNIPAm is insensitive to base, this composite hydrogel will shift into a helix when pH = 9 (Figure 5a bottom left). 

The multi-step photopolymerization based on the bonding of new monomers to the existing network makes it easy to integrate multiple stimuli-responsive hydrogels into one monolithic gel, which enables the selective shape transformation from a single flat sheet. Wang et al. also replaced PAA strips in the upper layer with poly(1-vinylimidazole-*co*-acrylamide) (P(VI-*co*-AAm)), which can swell as a response to acids while the bottom layer is kept unchanged. Consequently, by changing pH from 9 to 1, the formed helix can change its chirality, roll into a circular shape or flip itself without changing chirality according to the strip orientation in the top and bottom layers (Figure 5b). 

#### 2.2.2. Liquid Crystal Networks or Elastomers-Based Helical Structures

Liquid crystals (LCs) can flow as a liquid while preserving their crystalline molecular structure. This state of matter can not only be found in low-molar-mass chemicals that have revolutionized the display market, but also can be realized in polymeric materials such as liquid crystal polymer networks (LCNs) and liquid crystal elastomers (LCEs). For detailed information of the microstructure and classification of liquid crystal polymers, please refer to the excellent review paper by White and Broer [73].

LCNs or LCEs can change their shapes as a response to external stimuli such as temperature (thermotropic), light (phototropic), or solution concentration (lyotropic), allowing them to function as soft actuators for a wide range of potential applications such as medical devices, microfluidic and microelectromechanical systems, artificial muscles and soft robotics [74,75,76,77]. Unlike the isotropic swelling or shrinkage of hydrogels, LCNs or LCEs can undergo anisotropic deformation that originates from their heterogenous microstructures, in which the chain-like molecule has certain preferential alignment directions. When subjected to external stimuli, the molecular alignment will deviate from the original direction, leading to macroscopic expansion perpendicular to the molecular orientation and contraction along the molecular alignment. This anisotropic response is inherent in LCNs or LCEs microstructures without the introduction of additional reinforcement or the integration of different material compositions, which is a distinct advantage compared to hydrogel systems. Recent progress on designing programmable self-morphing LCNs or LCEs materials can be found in these extensive reviews [78,79,80,81,82,83,84].

**Figure 6:**
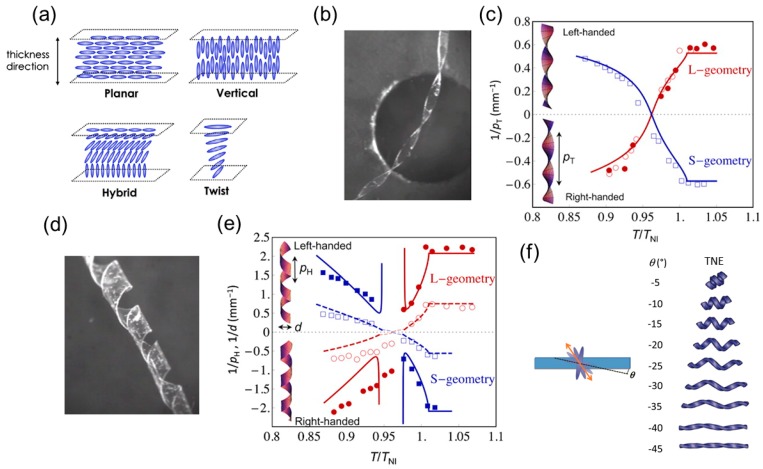
Nematic configurations and formation of helix/spiral induced by temperature variation. (**a**) Planar-, vertical-, hybrid-, and twist-nematic configurations of LCNs; (**b**) Formation of a helicoid ribbon from narrow TNE film at 330 K; (**c**) Inverse of the twist pitch (1/p_T_) as a function of normalized temperature (T/T_NI_, where T is temperature and T_NI_ is the nematic-isotropic transition temperature). Positive and negative p_T_ indicate left- and right handedness, respectively. Red circles and blue squares represent data of L- and S-geometry, respectively. Filled symbols indicate data obtained in cooling processes and open in heating processes. Theoretical predictions are represented by lines; (**d**) Formation of a spiral ribbon from the wide TNE film at 336 K; (**e**) Inverse of the helical pitch (1/p_h_) and the diameter (1/d) as a function of T/T_NI_. Positive and negative p_H_ indicate left- and right handedness, respectively. Red circles and blue squares represent data of L- and S-geometry, respectively. Filled symbols are data for 1/d and open symbols are data for 1/p_h_. Theoretical predictions are represented by lines; (**f**) Various simulated helical shapes corresponding to different off-axis angles. Simulation performed by Vianney Gimenez-Pinto. (θ). Figures reprinted from: (**a**) [85], with permission from Elsevier; (**b**–**e**) [31]; (**f**) [32], with permission from the American Physical Society.

For LCNs or LCEs, the shape transition is dictated by the programmable molecular orientation inside the matrix. By orienting the directors along the thickness of the LCN or LCE film, various types of LCNs or LCEs can be achieved such as planar-, vertical-, hybrid-, and twist-nematic configurations (Figure 6a). A uniform planar or vertical alignment will lead to a purely planar expansion or contraction while a nonuniform distribution of the director orientation such as the hybrid or twist alignment will buckle the flat sheet into 3D configurations. Urayama summarized studies on thermally triggered deformations including spiral and helix formation that arise from these different types of director configurations [85].

Among these different microstructures, twist-alignment LCNs or LCEs in which the director rotates left- or right-handedly by an angle (usually 90°) from the bottom to top surface can generate a helical shape in response to stimuli, and this actuation behavior resembles seedpod chiral opening with many similar behaviors. 

In the following sub-sections, we focus on the helical structures made of LCNs or LCEs that mimic *Bauhinia variegata*’s pod opening in terms of the responsive stimuli ranging from the most widely used ones, heat and light, to less-frequently used ones including chemical, humidity, and water. 

##### Thermally Responsive LCNs or LCEs

Heat is the most common stimulus for polymeric LC materials by far in that it can trigger deformation without any additive doped into the material. This thermal response is anisotropic owing to the different thermal expansion coefficients along different directions in the polymer. For example, upon heating, the thermal expansion coefficient along the molecular director is negative while it is positive perpendicular to the molecular direction in LCNs or LCEs [73,76,86]. By varying the molecular orientation inside one single LCN or LCE sheet, we can trigger the differential thermomechanical responses when exposed to external signal. This internal heterogeneity can be achieved in a single structure with one-step fabrication. This removes the need to combine different materials in one structure using multi-step synthesis, such as in the fabrication of multi-layer structures with layer-by-layer deposition.

Sawa et al. studied how the microscopic chiral alignment of mesogens in twisted-nematic-elastomer (TNE) films transfers to the macroscopic formation of helical ribbons [31]. They created TNE films with the director left-handedly rotating by 90° from the bottom to the top surface by photopolymerizing the mesogenic monoacrylate and crosslinker in a nematic solvent with chiral dopants. The mutually perpendicular directors at the top and bottom surfaces are formed by corresponding substrates coated by a uniaxially rubbed polyimide layer. They cut out the long ribbon specimen from the film in a way that the director at the middle plane is either parallel to the long axis (L-geometry) or the short axis (S-geometry) of the ribbon.

Through both experimental observation and theoretical modeling, they found that for both the S-and L-geometry, the width of the specimen determines what shape it forms. A narrow ribbon forms a helicoid (Figure 6b) and a wide one forms a spiral ribbon (Figure 6d). This behavior resembles that of a seedpod and can be explained by the domination of the bending energy (in the narrow case) or the stretching energy (in the wide case) as discussed in Section 2.1. Quantitative models capturing the transition from helical to spiral structure in a TNE ribbon have also been proposed [87,88]. In the narrow case, temperature variation will change the twist pitch (p_T_) of the helicoid (Figure 6c). In the wide case, it will change the helical pitch (p_H_) and diameter (d) of the spiral ribbon (Figure 6e). In both cases (Figure 6c,e), handedness reversions are involved and the shape changing processes are thermally reversible. The structural parameters (e.g., p_T_, p_H_, and d) of the TNE ribbons are no longer dependent on temperature variation once it is greater than the nematic-isotropic transition temperature (T_NI_ = 367 K) because it has become the isotropic phase.

Intuitively, one might ask what shapes TNE ribbons will form if the director at the mid-plane of the film is neither along the long nor the short axis of the ribbon (i.e., not S- or L-geometry). Sawa et al. examined the shape evolution of off-axis TNE ribbons as a function of temperature using experiments and finite element analysis (FEA) [32]. They showed that when the director at the mid-plane is off axis, the ribbon will not shape into helicoids but into distorted spiral ribbons. They predicted the varying spiral shapes with respect to different off-axis angles (θ) using FEA simulation (Figure 6f). 

The formed helix/spiral shape of the TNE will return to its original shape immediately upon removal of the heat source. Lee et al. designed an autonomous shape fixing procedure that retains the spiral ribbon structure of a TNE film even after the heat stimulus is removed [33]. This hands-free shape fixing ability is obtained by rapid cooling. In a dynamic mechanical analysis, they demonstrated that this technique retains up to 96% of the strain (an indicator of deformation) in the shape after cooling. Furthermore, if the formed shape is under an external mechanical constraint (e.g., tweezers and adhesions) the cooling rate can be moderately slower. 

By using photopatterning techniques, spatial variation of the domain orientation in monolithic TNE films [33] and dual-phase (nematic and isotropic) monolithic LCE films [37] have been fabricated to form helical shapes. The shape transformation can also be achieved by assigning LCE films with different chemical compositions or nematic configurations [34]. Finally, instead of using single-layer twist nematic LCNs, LCEs are used in bi-layer structures to form helical/spiral ribbons. Agrawal et al. have constructed a LCE-polystyrene bilayer, where the polystyrene (serving as the constraining layer) is deposited on the LCE film with a certain angle and curls into a helical shape upon heating [35]. Boothby and colleagues have also designed a bilayer structure that is composed of a layer of hydrophilic polymer (serving as the constraining layer) and a layer of LCE [36]. As temperature increases, the LCE layer stretches in the direction perpendicular to the molecular director which is 45° to the long axis of the LCE ribbon, leading to a helicoid. When the temperature drops to room temperature, a spiral ribbon is formed.

##### Light-Responsive LCNs or LCEs

Light is another attractive source of stimulus to induce a macroscale mechanical response of LCNs or LCEs because it is readily available, versatile, controllable and of high spatial resolution. An increasing number of studies have investigated the conversion of light into motion of LC-based soft actuators [73,74,89]. We only focus on those works that produce motions mimicking the opening of seedpod (i.e., the formation of a 3D helical/spiral structure from a 2D ribbon). 

A detailed preparation protocol for a LCN-based photo-responsive soft actuator is described by Iamsaard et al. [90]. Briefly, azobenzene is introduced into the LCN’s covalent structure to serve as a molecular photochromic switch. The host matrix is a mix of low molecular-weight nematic LC, acrylate-functionalized nematic LC, and a photoinitiator. The mixture is doped with chiral dopants that generate a 90° smooth twist between the top and bottom surfaces. 

**Figure 7:**
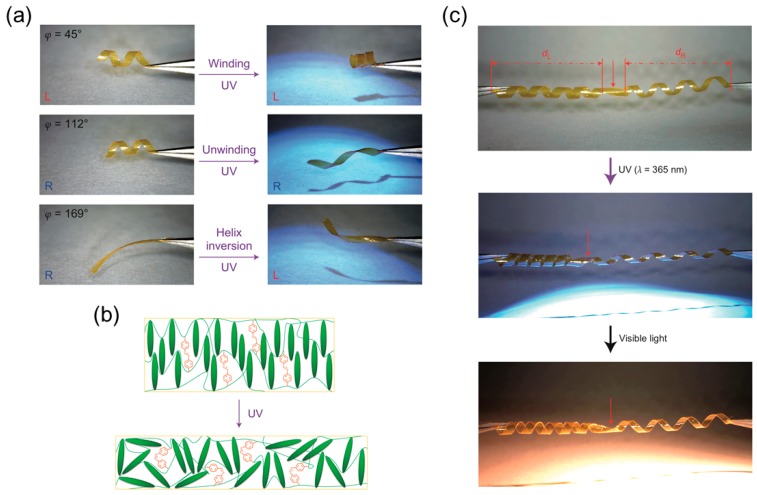
Light-induced helical motion of a LCN ribbon. (**a**) Change in pitch and inversion of handedness of spiral ribbons cut at different angles (φ) irradiated by UV light; (**b**) Anisotropic deformation at the molecular level: shrinkage along the director and expansion in the direction perpendicular to the director; (**c**) A proof-of-principle for an actuator capable of performing complex motion: the kink in the middle connecting helices of opposite handedness shows a smooth push-pull motion. Figure reprinted from [38] by permission of Springer Nature.

The formation of a helical shape of this material induced by ultraviolet (UV) light was observed in 2005 [91]. Later, researchers deliberately studied the winding and unwinding motion of the helix formed by LCN ribbons [38]. As shown in Figure 7a, in addition to the findings on how the ribbon cutting angle (φ) determines the pitch and handedness of the helical shape, Iamsaard and colleagues have some interesting new discoveries. They observed that under irradiation with UV light the helical pitch decreases in left-handed ribbons and increases in right-handed ribbons and therefore large contractions and elongations of the ribbon were generated. They also witnessed the inversion of the helix from right-handed to left-handed when the ribbon was irritated by UV light at large cutting angle. Similar to the mechanism of deformation induced by heat, these shape changes of LCN with photoresponsive dopants irradiated by light are consequences of anisotropic deformation at a microscopic level: molecules contract along the director and expand in perpendicular direction (Figure 7b). The authors demonstrate a proof-of-concept of a photomechanical actuator in response to alternating UV and visible light. This actuator consists of an LCN-based ribbon with opposite handedness on two sides connected by a kink that performs continuous push-pull motions like a piston (Figure 7c), and this conversion of handedness in the synthesized LCN resembles the perversion found in tendrils or other plants. Inspired by this, a biomimetic bilayer soft actuator fabricated by Wang et al. is developed using LCE that can perform chiral twisting motions when irradiated by UV [39]. The top and bottom ribbon layers have different photo-responsive properties and are glued to each other with a tilted angle, and this angle determines the chirality of the formed helix.

##### Other Stimuli-Responsive LCNs or LCEs

Other stimuli that actuate LC-based shape transformations also have caught researchers’ attention in recent years. We briefly review several examples of actuations by chemical-, humidity-, water-, and magnetic-stimuli. 

**Figure 8:**
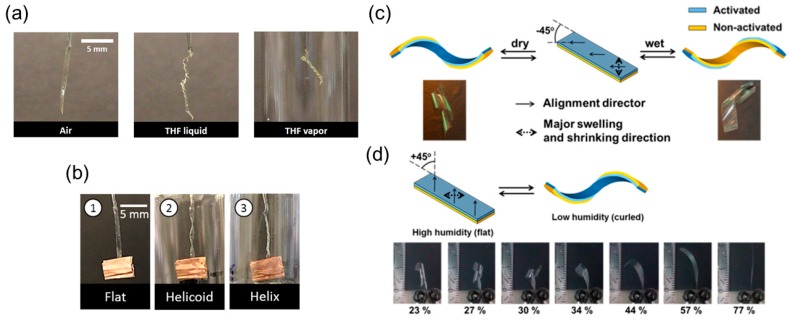
Formation of a helical shape triggered by other stimuli. (**a**) TNE ribbon in air, THF liquid, and THF vapor; (**b**) TNE ribbon remaining flat in air and curling into a helicoid and a self-contacting helix in THF vapor as a function of time; (**c**) A bilayer LCN ribbon, in which the director is 45° to the long axis of the ribbon, showing left-handedness when dried and right-handedness when wet; (**d**) A bilayer LCN ribbon, in which the director is −45° to the long axis of the ribbon, exhibiting a smooth transition in shape from flat to curled as humidity decreases. Figures (**a**,**b**) reprinted from [40] with permission from Elsevier; (**c**,**d**) from [41], with permission from the American Chemical Society.

Boothby et al. have examined LCEs as chemoresponsive actuators [40]. They experimentally showed that TNE ribbons form into twisted shapes in response to tetrahydrofuran (THF) as the chemical stimulus in liquid or in vapor form (Figure 8a). This TNE can even carry a weight 100 times heavier than itself and twist into a helicoid and a helix as a function of time (Figure 8b). 

Humidity in air has proven to be an effective controlling parameter to determine the extent of curling of an LC polymer. Instead of using a monolithic TNE ribbon, a bilayer planar aligned LCN ribbon is fabricated by De Haan et al. [41]. The two layers are attached to each other in a way that the angle between their directors and the long axis of the ribbon is −45° (Figure 7c). When the ribbon is wet on the activated side, it curls into a right-handed helix, and when it is dried, it reverses to a left-handed helix. When the angle is 45°, however, the ribbon is flat when it is exposed to high humidity and right-handed when exposed to low humidity. This asymmetry is achieved by locally converting the uniaxially aligned LCN film to a hygroscopic polymer salt that swells perpendicular to the director. It is also shown that the extent of curling of the specimen depends on the humidity level that it is exposed to (Figure 8d). 

**Figure 9:**
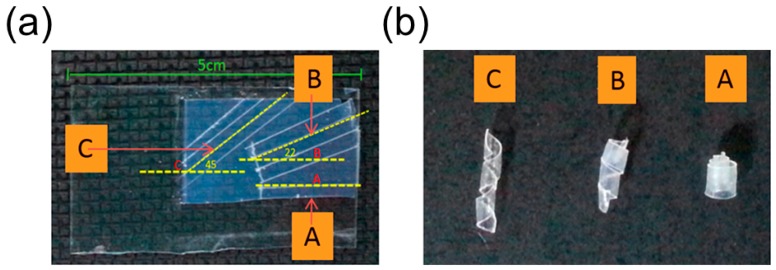
Formation of a helical shape triggered by water/acetone. (**a**) Ribbons cut at different angles (A: 0°; B: 22°; C: 45°) on a single-layer LCE film where the director is in the horizontal direction; (**b**) Formation of different helically coiled shapes of A, B and C in response to water exposure. Figure reprinted with permission from: (**a**,**b**) reference [42], American Chemical Society.

A single-layer actuator based on LCE that changes shapes in response to water/acetone is designed by Kamal and Park [42]. The asymmetric deformation (including forming a spiral shape) of the LCE-based ribbon mainly arises from the nematic-isotropic transition and a porous gradient in the thickness direction (i.e., during photopolymerization, the UV-exposed side is smooth while the other side is highly porous). After a straight ribbon is cut from the LCE film and immersed in water, it will form a spiral shape of a certain pitch depending on the angle between the long axis of the ribbon and the director’s orientation of the LCE (Figure 9a,b). This process is reversed when the curled ribbon is immersed in acetone. 

Lastly, via incorporating magnetic nanoparticles in LCE-based materials, soft actuators stimulated by magnetics field can be obtained [92,93,94], offering a practical method to remotely control the flat-to-helix shape transformation without direct contact with the external stimuli.

#### 2.2.3. Shape Memory Polymers-Based Helical Structures

Shape memory polymers (SMPs) that can switch from a temporary state to a permanent state under external stimuli such as heat [95] and water [43] are widely used in actuation systems since the shape transformation is usually predictable and easily programmed [96,97]. Although shape memory polymers have a wide variety of possible shape-change mechanisms, they generally involve a glass transition temperature *T_g_*. Below this temperature, the polymer is stiff and glassy, while above it the polymer is rubbery and can flow to relax the internal residue stress. Typically, to employ the memory effect of SMPs to achieve actuation, one needs to deform the initially stress-free shape memory polymers and then decrease the temperature below *T_g_*, during which the deformation needs to be held at all time. After this shape fixing stage, due to the interaction between polymer chains, SMPs cannot recover to the stress-free shape. When the temperature is later increased above *T_g_*, the polymer chains slip with each other like rubble, the internal stress is relaxed, and SMPs deform back to their permanent configurations [98].

**Figure 10:**
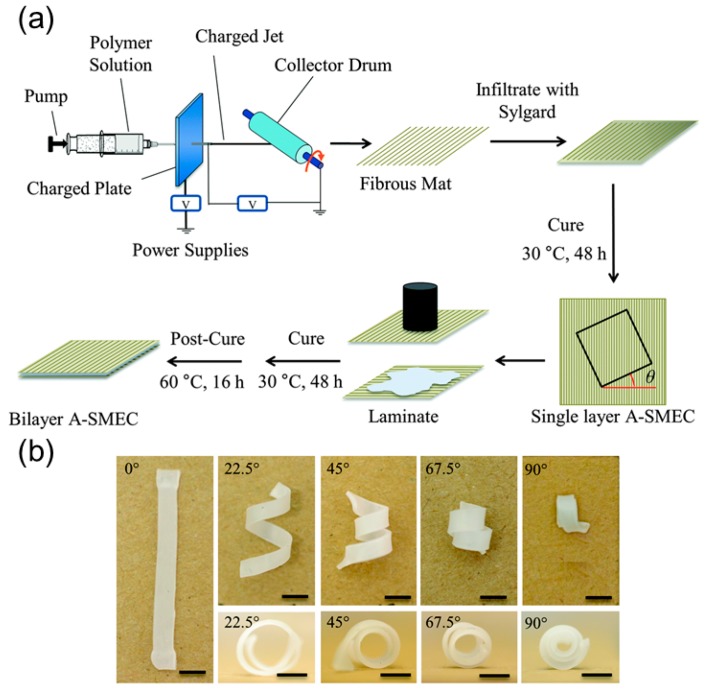
Shape memory polymers-based helical structures mimicking *Bauhinia variegata*. (**a**) Schematic illustration of the fabrication process of the shape memory elastomeric composite; (**b**) Experimental images of the coiled bilayer composites after heat treatment. The left-top corner of each image shows the tilting angel and the scale bar is 4mm. (**a**,**b**) are from [44], reprinted with permission from The Royal Society of Chemistry.

Based on the shaping fixing and recovery of SMPs, Robertson and coworkers [44] fabricated a shape memory laminated composite that can coil into a helix upon heating. During the fabrication process, they first electrospan aligned poly (vinyl acetate) (PVAc) fibers guided by the electric filed and then infiltrated the fiber’s interspace with poly(dimethylsiloxane) (PDMS) precursor (Figure 10a). A single-layer elastomeric composite reinforced with unidirectional fibers is obtained after curing process, which is later used to fabricate a bilayer mat by adhering two single-layer laminas. The adhesion is realized via curing the uncrosslinked PDMS, and the tilting angle between the fiber’s directions in two layers can be tuned before the stacking step (Figure 10a).

The shape memory effect of this composite relies on the shape recovery of the PDMS matrix and shape fixing ability of PVAc fibers. PVAc fibers can develop plastic strain below *T_g_* and relax when the temperature exceeds the critical point. Therefore, mechanical stretching below *T_g_* of the laminate composite will produce plastic strain inside PVAc fibers while the PDMS matrix remains elastic. The differential responses of PVAc fibers and PDMS matrix during the strain relaxation will uniaxially deform the matrix along the fiber’s alignment. Owing to the tilting angle between fiber reinforcements in two layers, the flat composite sheet coils into a spiral shape like the pod opening of *Bauhinia variegata*. The authors also found that the variation of the tilting angle will change the pitch and radius of the spiral shape, sharing a similar behavior with other synthesized helical structures (Figure 10b).

## 3. Helical Structures Mimicking Tendril’s Coiling

### 3.1. Coiling Mechanism of Tendrils

In addition to the chiral pod’s opening of *Bauhinia variegata*, another vivid example in the plant kingdom which exhibits helical shape evolution is tendril’s coiling, whereby an initially straight tendril can not only coil into a spiral shape as it grows [12] but also develop co-existing left-handed and right-handed segments connected by perversions for enhanced structural support [13].

**Figure 11:**
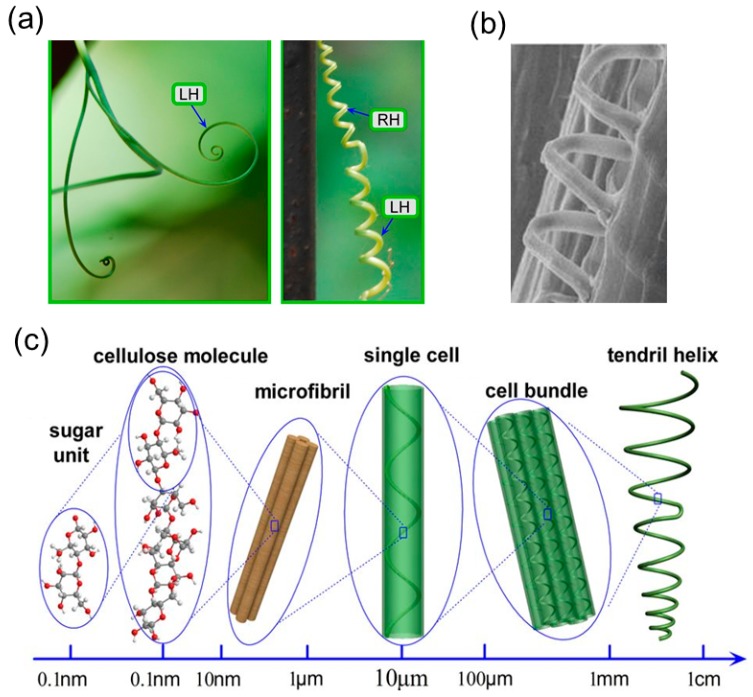
Coiling mechanism of *Towel Gourd* tendrils. (**a**) *Towel Gourd* tendril coils into a spiral shape with left-handedness before it touches a support (left) and forms a perversion connecting the right-handed and left-handed sections once it attaches to a support (right). ‘LH’ and ‘RH’ represent left-handed and right-handed, respectively; (**b**) Image of helical cellulose fibril inside cell’s matrix under scanning electron microscope; (**c**) Hierarchical chirality inside *Towel Gourd* tendril from the molecular level to the macroscopic shape. (**a**–**c**) are from [12], reprinted with permission from Nature Publishing Group.

The tendrils can have two different stages of growth. Initially, the tendril is soft and flexible. The tip of the tendril will follow a spiral trajectory before it touches a support [14] (Figure 11a, left). During this growing stage, this rotational growth can be attributed to the internal chiral building blocks. Specifically, for *Towel Gourd* tendrils, the chiral cellulose molecules aggregate to form the helical cellulose fibrils inside the cells (Figure 11b). During biological growth, the helical angle of the cellulose fibrils will be adjusted and introduce an internal torque on the fiber-like cell [99], and the twisted cell will create the macroscopic coiling morphology of the tendril [100]. This bottom-up hierarchical chirality transfer covers different length scales from the molecular level to the macroscopic level (Figure 11c) and a similar mechanism has been discovered to contribute to the helical morphology in other species, such as hygroscopic coiling of the stork’s bill awns (*Erodium gruinum*) [101] and bacterial flagella [102]. 

When the soft tendril attaches to a support, it enters a new growing period during which the tendril becomes woody and strong, and an intrinsic curvature is formed due to differential growth [2]. In the cucumber tendril, once the tendril touches a support, the ventral cells undergo a more severe lignification than the cells in the outer layer. This differential lignification leads to the asymmetry contraction of the ribbon-like fibers inside the tendril, and thus an intrinsic curvature emerges to accommodate this misfit length variation and further coils the tendril [13]. A perversion always appears in this growing stage. Here, the term ‘perversion’, which describes an inversion linking segments of the opposite chirality, was used by German mathematician Listing, and the observation and study of its existence in climbing plants dates back hundreds of years to the age of Charles Darwin. This mirror-symmetry morphology is also observed in other biological and artificial systems, such as the human umbilical cord, gut’s looping [103], leave’s edge [104], bacterial flagella [1], and strained nanoribbons [105]. For the related history on tendril’s perversion, one can refer to Goriely’s book [14].

The emergence of perversions in tendrils can be explained using a mechanical analogy—an elastic rod with an intrinsic curvature. This elastic rod will assume the ring shape at rest but will become straight if enough tension is applied at both ends. One can first straighten the rod and then gradually decrease the tension until the tension is below the critical point, and then the straight rod begins to coil owing to bending instability [15]. A helical shape is a stable solution for the morphology near two ends under this circumstance using Kirchhoff rod theory. Due to topological constraints, the helical shape cannot maintain the same chirality along the entire length. Thus, each half of the rod coils into a helix with opposite chirality, with a perversion connecting the two.

Unlike the hierarchical chirality transfer mechanism in the initial growing stage, the coiling of tendrils upon touching the support relies on the emergence of intrinsic curvature as well as the boundary conditions. This new dominating mechanism is simple yet inspiring because a helical shape with perversions is a ‘twistless’ spring and thus a good example of an ideal Hookean structure—the tension force is linear with elongation. The traditional helical spring is only linearly elastic when elongation is small and gets strain-stiffening in the large elongation regime. The deviation from the linear Hookean property comes from the fact that the spring’s ends cannot freely rotate during the loading process. This problem can be overcome by utilizing a helical shape with perversions [15], and the underlying physics is illustrated as follows. For a helix with one perversion locating at its center, it can be treated as two helical springs connecting to each other via the perversion. During the loading process, the connecting ‘end’ of these two helical springs can freely rotate since the perversion can rotate around the helix’s axis, and hence this dual-chirality helical shape can retain the linear force-extension response in a larger elongation region compared to the common helical spring [106]. Therefore, the study on the formation of helical structures mimicking the tendril’s coiling is not only important in understanding the plant’s morphology, but will also be beneficial to the engineering applications such as nanomechanical systems. 

In the following sub-sections, we provide an overview on the helical structures mimicking the two growing phases of tendril’s coiling before and after its attachment to a support, respectively. In the first part, the helical structures with chiral building blocks, specifically carbon nanotubes (CNTs) in a helical arrangement, are introduced as representative examples inspired from the hierarchical chirality transfer mechanism. This type of helical structures can provide contractive and rotatory actuations under external stimuli, standing out as a strong candidate for light-weighted artificial muscle fibers. In the second part, the helical structures with perversions resulting from an intrinsic curvature in a ribbon or rod are presented. The mechanical properties of these engineering structures are also discussed.

### 3.2. Helical Structures with Hierarchically Chiral Building Blocks

Carbon nanotubes (CNTs) are well-known for their outstanding mechanical and electrical properties. Great efforts have been taken to assemble CNTs into 1D (fiber), 2D (film) and 3D (vertical aligned arrays) macroscopic elements without losing CNT’s excellent performance for applications [107]. Among these structures, the twist-aligned CNT pristine fibers [108] and their hybrid modifications by injecting functional polymers inside [109,110,111] have exhibited excellent performance as artificial muscle fibers. They are light-weight yet able to provide contractive and rotatory actuation when exposed to thermal, electric, or solvent signals [110]. Taking thermal actuation as an example, the aligned CNTs will contract along the axial direction and expand along the radial direction when the temperature rises, and this anisotropic response will untwist the helically aligned CNTs in the fiber, yielding a reversible torsion motion [112]. If the straight CNT fiber further coils into a spring under extreme torsion, the twisting and untwisting of the local section will be converted into elongation and contraction of the entire structure along the axial direction, which is similar to the stretching and compressing of an elastic spring.

Inspired by the hierarchical structure in *Towel Gourd* tendrils, Peng’s group improved the performance of single pristine CNT fibers by assembling multiple CNT fibers together and twisting them into a compacted, coiled, helical structure [113]. These hierarchical helical fibers (HHFs), like the constitutive single CNT fibers, can contract and rotate under different stimuli such as vapor [113] and electric current [114], and show better properties in terms of maximum contractive stress, working life, and rotatory speed compared to the single CNT fibers (Table 2). It is believed that this outstanding stroke output originates from multi-level gaps and helical turns stored inside the HHFs, resembling the bottom-up chirality transfer in many biological systems.

**Figure 12:**
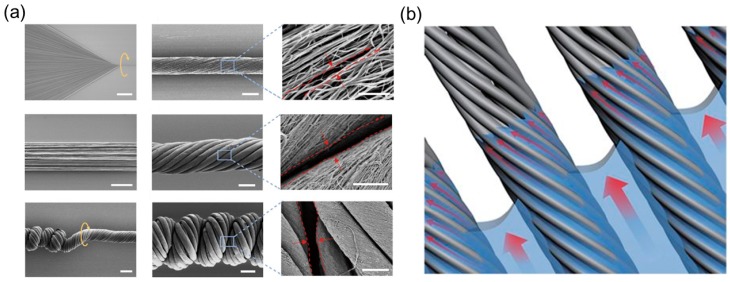
CNT-based helical structures mimicking *Towel Gourd* tendrils. (**a**) Groups of scanning electron images showing the fabrication process of the hierarchical helical fibers based on twisting MWCNTs. first row: dry-spinning (left, scale bar 500 μm, primary fiber (middle, scale bar 10 μm) and the nanoscale gaps between MWCNTs (right, scale bar 500 nm). Second row: bundle of primary fibers (left, scale bar 200 μm), twisted primary fibers (middle, scale bar 30 μm) and the microscale gaps between primary gaps (right, scale bar 2 μm). Third row: coiling of multi-ply primary fibers when twisting exceeds the threshold (left, scale bar 50 μm), hierarchical helical fiber (middle, scale bar 30 μm) and gaps inside HHF (right, scale bar 10 μm); (**b**) Hierarchical gaps, including microscale gaps between primary fibers and nanoscale gaps between MWCNTs, facilitate the solution’s infiltration; (**a**) is from [115], reprinted with permission from Nature Publishing Group; (**b**) is from [113], reprinted with permission from Nature Publishing Group.

The fabrication process utilized the so-called ‘writhing instability’ in the elastic rod—a straight rod will coil itself if the torsion applied on its ends exceeds a critical value [15]. Here, Peng’s group first synthesized the multiwalled carbon nanotube (MWCNT) arrays using chemical vapor deposition and dry-span the MWCNTs into a primary fiber. The MWCNTs formed helical arrangement inside the primary fiber with nanoscale gaps between them (Figure 12a, first row). Then multiple primary fibers were stacked together and twisted (Figure 12a, second row). Once the applied twisting reached a critical value, the bundled primary fibers coiled compactly along the axial direction into the secondary fibers (Figure 12a, third row). During this twisting treatment, the primary fibers took the helical arrangement with microscale gaps between them. Obviously, several helical configurations exist in secondary fibers sweeping different length scales—the nanoscale helical MWCNTs in the primary fibers, the microscale helical primary fibers in the secondary fibers, and even the macroscopic helical shape if the straight secondary fiber further coils into a spring based on the thermal setting process using a mandrel.

The fabricated secondary fibers can contract or rotate when exposed to a polar solvent or its vapor. The underlying mechanism originates from the internal structure where gaps provide space for solution infiltration, expanding the structure and initiating the motion as a result. The experiments show that the secondary fibers have better performance than the primary fibers in terms of maximum contractive stress and rotatory speed etc. (Table 2), which can be explained from two perspectives. First, the secondary fiber contains more helical turns than the primary fibers because of an additional step of twisting, which in turn gives more contractive and rotatory actuations induced by the local untwisting. Second, the co-existence of nanoscale gaps between the MWCNTs and microscale gaps between the primary fibers facilitates the fast infiltration of the polar solution (Figure 12b). Accordingly, the rotatory speed of the secondary fiber is higher than that of the single primary fiber since the primary fiber only contains nanoscale gaps between the MWCNTs (Table 2). Periodic contractive and rotatory motion can be realized by continually applying and withdrawing the external stimuli, and it is demonstrated that the hierarchical helical structures inside the secondary fiber can stabilize the fiber’s integrity and prolong its working life. Evidence is shown in Table 2. The secondary fibers composed of 20 primary fibers can maintain reversible actuation without obvious fatigue after 20 cycles of ethanol infiltration, whereas the performance of the single primary fiber decreases significantly after a 15-cycle loading due to the partial untwisting of stacked MWCNTs.

**Figure 13:**
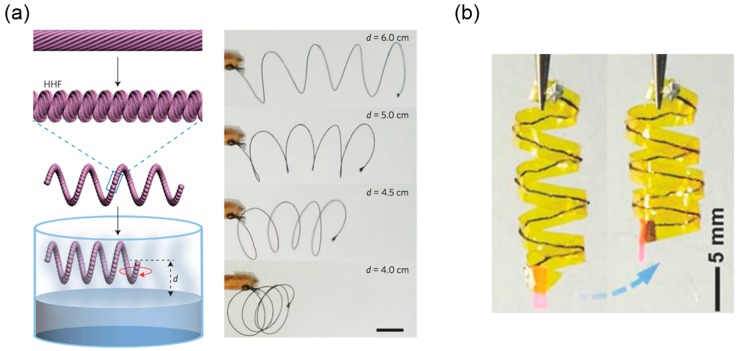
Contractive actuation of the coiled secondary fibers made of MWCNTs under vapor and electric current stimuli. (**a**) The contraction actuation of the hierarchical helical fiber when getting close to the dichloromethane (left: schematic; right: experiments). *d* is the distance between the spring and liquid surface (scale bar 2 cm); (**b**) Electromechanical contraction actuation of a left-handed Kapton film with HHF inside; (**a)** is from [113], reprinted with permission from Nature Publishing Group; (**b**) is from [114], reprinted with permission from 2015 Wiley.

One potential application of these hierarchically structured fibers is in vapor-detecting sensors. If one coils the straight secondary fiber into a helix, this spring will contract when it gets close to the liquid surface of certain polar solvents. For example, the contractive strain will increase from 0% to almost 60% when the distance between the spring and the dichloromethane’s surface (*d*) decreases from 7.5 cm to 6 cm and gets further increasing if *d* is shortened to 4.0 cm (Figure 13a). Reversible contraction is maintained after 50 cycles of exposure.

The hierarchical CNT fibers can also contract and rotate in response to electric current. Peng’s group explained it as the result of electromagnetic interaction between aligned CNTs based on Ampere’s law [116,117], whereas another plausible interpretation is given as the indirect thermal response to Joule heating generated by current passing through CNTs [112]. Nevertheless, like the vapor-triggered actuation, the electromechanical performance of the secondary fiber is better than that of the single primary fiber, and it is probably attributed to the multilevel internal construction. For example, additional electromagnetic interaction can exist between the neighboring primary fibers in addition to the interaction among the CNTs inside a secondary fiber. Relying on the high fracture strain (245%) and stress (220MPa), Peng’s group also sewed the fiber into a Kapton film and used the heat-setting process to transform the straight fiber into a 3D helix. The helical integrated structure will wind or unwind itself when it receives an electrical signal, which mimics the tendril’s coiling process (Figure 13b).

### 3.3. Helical Structures with Perversions

Tendrils use another strategy to coil after grabbing supports other than the hierarchically chirality transfer—they produce an intrinsic curvature by differential growth of their cells, and a perversion will form in this stage to connect helices with opposite chirality. This mechanism can be reproduced in artificial systems by combining two layers with different natural lengths into one integrate rod-like or ribbon-like structure. Here, the ‘natural length’ represents the length when the layer is stress-free alone. To accommodate the misfit between the natural lengths of two layers, the composite structure will bend into a certain curvature at rest.

Two common methods can be used. One can mechanically stretch or compress a layer and then attach it to another passive layer. Gradually reducing the pulling force or shortening the distance between two ends will coil the straight bilayer structure with the emergence of one or multiple perversions. The other strategy is to combine two layers with different responses to the external stimuli. The initially straight bilayer structure will spontaneously form an intrinsic curvature once exposed to the external stimuli, since the two layers undergo different extents of swelling or shrinkage. One feasible technique to fulfill this goal is to electrospin bi-component fibers with sub-microscale diameters, paving the way to designing biomimetic helical structures at the nanoscale.

#### 3.3.1. Bilayer Elastomers with Misfit Natural Length

**Figure 14:**
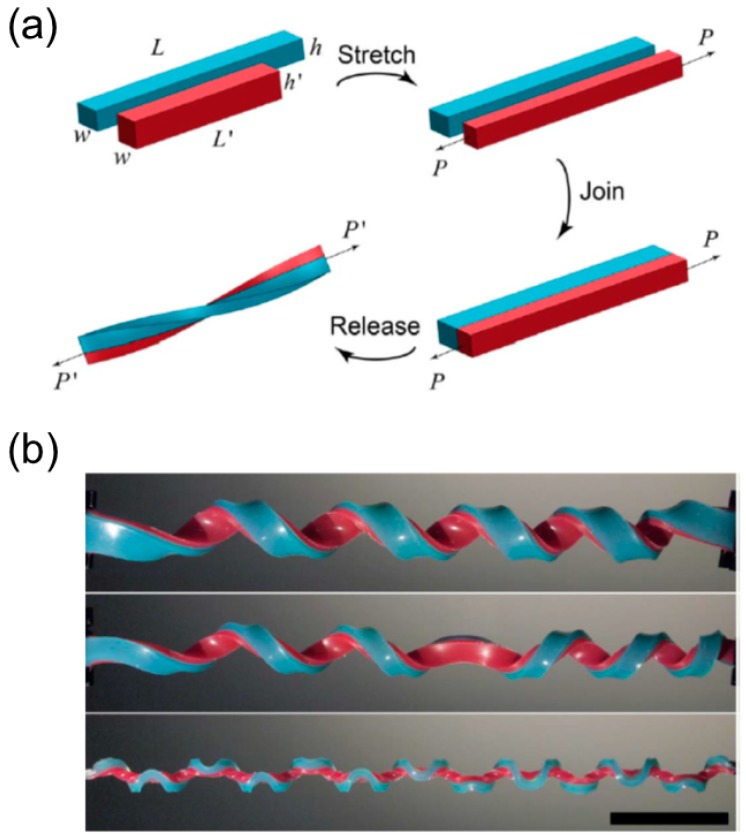
Formation of multiple perversions in a bilayer elastomer system. (**a**) Schematic illustration of the fabrication process of a bilayer elastomer with the misfit natural length; (**b**) The perversion’s number increases as h/w decreases (h/w= 4 (top), 2.7 (middle), 0.83 (bottom)); (**a**,**b**) are from [118], reproduced with permission, copyright: © 2014 Liu et al.

One can pre-stretch an elastomer strip and attach it to the other undeformed elastomer layer to generate the intrinsic curvature in a bilayer strip with both ends held [13,118,119]. The schematic illustration of the fabrication process is shown in Figure 14a. 

A short strip (red) with length $L^{\prime }$, width $w^{\prime }$ and thickness $h^{\prime }$ is stretched to the same length *L* as the long strip (blue) and bonded to the long strip side-by-side. The two strips have the same width *w* and thickness *h* after stretching. The two holding ends are gradually released with or without the end’s rotating, and out-of-plane bending and twisting will occur once the distance is shortened below the critical value. This fabrication prototype is simple and thus adopted by many researchers to perform systematic investigation on such unique mechanical system [13,118,119], leading to the discovery of a variety of interesting properties belonging to a helix with perversions.

##### Emergence of Multiple Perversions

The first noteworthy property of this bilayer elastomer is that multiple perversions can emerge simultaneously once the pulling force decreases to the critical point, and this distinct shape is referred as a ‘hemihelix’ [118,119]. At the onset of this out-of-plane distortion, perversions appear separately along the strips and their locations stay unchanged in the rest of releasing, with their amplitudes growing. Huang et al. found that the number of perversions *N* is deterministic and only depends on several parameters including the pre-stretch ratio ($\chi =\left(L-L^{\prime }\right)/L^{\prime }$), the aspect ratio of the cross-section (h/w), and the length of the ribbon (*L*) [119]. A unified relationship based on the dimensional analysis is proposed as wN/L=f(χ, h/w) [118]. Qualitatively, when h/w is large, the bending is energetically preferred, and the strip will form a helix with no or a few perversions during the end’s releasing. As h/w increases, twisting becomes favorable while bending costs more energy, and alternatively the strip will twist as well as bend to give rise to more perversions (Figure 14b). 

The perversion’s number is irrelevant to the loading process, such as loading speed and loading history. For example, repeatedly pulling the bilayer elastomer to be straight and then releasing it will not change the perversion’s number. In addition, although the rubble-like elastomers are always used as strips in experiments, finite element analysis demonstrated that the constitutive behavior of the materials only have negligible effect on the perversion’s formation [119]. The perversion’s number almost keeps the same when the material property is changed from hyper-elastic to linear elastic material. The perversions are located separately along the bilayer ribbon as if they repel each other, reducing the interaction energy between them [120]. 

##### Buckling Instability

Another significant observation is that multiple perversions can appear even if one strip’s end can freely rotate when it approaches the other. This unique property is beyond the scope of tendril’s coiling since it rules out the requirement of the topological constraint to create the perversion inside a helix. Pioneering research about tendril’s coiling assumes that the tendril’s ends are attached to the supports and cannot freely rotate. Therefore, the boundary conditions put a restriction such that the linking number should be invariant during the growing process. As a result, a 3D configuration in which one perversion links two helices with opposite chirality becomes the plausible solution because it satisfies the boundary conditions and reduces the total elastic energy as well [2,15]. However, from experiments, theoretical analysis and finite element simulation of this bilayer elastomer system, it is discovered that the occurrence of perversions results from a buckling instability at the early stage of release. Multiple buckling modes can be excited almost simultaneously and compete since their critical points in terms of the stretch ratio χ are closely spaced. The helix configuration without perversions stays at the lowest energy level, but the amplitudes of other modes with multiple perversions can grow faster and dominate the following shape evolution when the aspect ratio h/w is small, leading to the formation of hemi-helices [118].

##### Over-Winding

**Figure 15:**
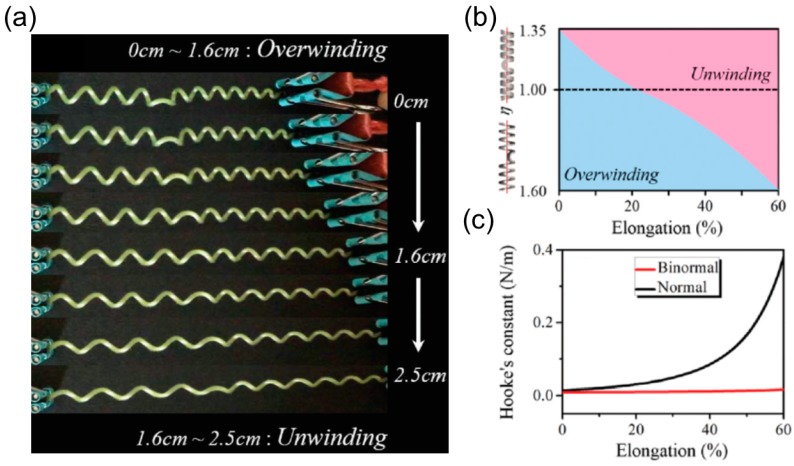
Mechanical properties of a helix with one perversion. (**a**) The tendril exhibits over-winding initially and then unwinds itself during pulling; (**b**) The phase diagram separating the unwinding and over-winding regimes in terms of elongation and η; (**c**) The Hooke’s constant of a scrolled SiGe/Si/Cr nanohelix with the normal or binormal cross-section under extension. (**a**–**c**) are from [106], reproduced with permission from The Royal Society of Chemistry.

The third fascinating behavior of a helix with perversions is over-winding during the pulling process. As shown in Figure 15a, a coiled tendril with one perversion in the middle is stretched along the axial direction. Intuitively, one would assume that the tendril will untwist itself until it gets straight. However, this is not always true. At the low elongation range (0–1.6 cm), the tendril further coils itself by adding more turns on both sides of the perversion. Only at high elongation range (1.6–2.5 cm) does the tendril untwist and get straight when the pulling force is strong enough.

Over-winding results from the competition between twisting and bending and it can be quantitively measured as the ratio of bending stiffness *B* to the torsion stiffness *C* of the cross section (Γ=B/C=1+ν for the circular section made of isotropic elastic material, ν is the Poisson’s ratio). When the bending stiffness is high compared to the torsion stiffness, this slender structure tends to maintain the intrinsic curvature since bending costs more energy. Alternatively, it chooses to change its pitch and radius by twisting itself to accommodate the axial extension. Note that this twisting-dominating ‘strategy’ is accessible because the perversion can rotate around the axis, enabling the connecting ‘end’ of the helix in each half to freely rotate.

The relative magnitude of the bending stiffness and torsion stiffness Γ can be adjusted by many methods. Gerbode et al. embedded inextensible fibers in the interior part of the cross section and added incompressible copper wires to the exterior side, inspired by the microstructure of the cucumber tendril [13]. This reinforcement increases the bending stiffness and induces over-winding behavior. For a rectangular cross section made of isotropic elastic materials with width *w* and thickness *h*, the ratio Γ depends on the width-to-thickness ratio η=w/h. The higher η is, the lower Γ will be. Dai et al. [106] employed the extensible rod theory to investigate the effect of η on the rotatory motion of a helix with one perversion during the pulling process. A phase diagram separating the over-winding and un-winding regimes is presented in terms of the axial elongation and η (Figure 15b). A helix with a normal section (η<1) is easy to over-wind itself while a helix with a binormal section (η>1) is inclined to exhibit unwinding phenomenon. The phase diagram also shows that a helix with one perversion will ultimately un-wind itself when the elongation is large enough regardless of the cross-section’s shape. This trend lies on the fact that the bending stiffness is large yet finite. Therefore, this slender structure cannot resist bending when the pulling force is extremely large and the helix eventually becomes straight.

##### Linear or Nonlinear Force-Extension Relationship

For a helical spring made of isotropic elastic material, it is stiffened quickly under extension and only stays linear in a small extensional range because the spring cannot untwist itself when ends are prohibited from making turns under pulling. This problem can be resolved by inserting a perversion inside the helix. During the pulling, the perversion can rotate around the axis, and equivalently the connecting ‘end’ of the helix in each half can make turns. As a result, this spring with dual-chirality in each half can exhibit a linear Hookean response in a large range of extension compared to the common spring [15]. 

Apart from increasing the extension range of linear response, the perversion also softens the helical spring as well. Gerbode et al. found that the stiffness of a perverted-spring is smaller compared to the helical spring if the perverted-spring shows unwinding behavior during pulling. A perverted-spring with over-winding behavior will first become softened and then get stiffened in the large extension region compared to the common helical spring [13]. Dai et al. [106] investigated the effect of η on the mechanical behavior of a helix with one perversion based on the experiments of the scrolled SiGe/Si/Cr nanohelix, and they observed that the binormal cross section (η>1) is a better choice to provide linear elastic response of the helix with dual-chirality than the normal cross section (η<1) (Figure 15c).

#### 3.3.2. Helical Rods from Electrospinning

Bonding two elastomer strips with different natural lengths can generate a perverted helix macroscopically, while creating a targeted shape in microscale or nanoscale requires more sophisticated methods. Electrospinning is a simple and established technique that can produce fibers with diameters ranging from hundreds of nanometers to a few micrometers [121]. It employs electric filed to drive charged polymer solution out of the syringe’s nozzle, first forming an elongated cone (Taylor’s cone) and then travelling as a thin thread in space once the electric force overcomes the surface tension. The ejected jet will hit the collector, and dry into a rigid fiber after the solution evaporates.

The electrospinning can produce fibers with heterogeneous structures. Methods include extruding different polymer solutions together out of the nozzle, modifying a homogeneous fiber selectively based on the light-triggered reaction and ejecting some certain anisotropic materials such as liquid crystalline cellulose. If the components in the heterogenous fiber swell or shrink differently during the solution’s evaporation, intrinsic curvature will appear and coil the initially straight fiber into a helix with perversions. This coiling mechanism is different from the other coiling effects during electrospinning, for instance, the ‘whipping’ instability during the jet’s moving [121] and the liquid rope-coiling effect when the polymer jet hits the collector [122], and will be the focus of the following context. Several cases that exploited the electrospinning to fabricate a perverted helix in micro- or nanoscale are presented based on the fabrication techniques. The mechanical properties of the mat made of the aligned, electrospun helical fibers are discussed.

##### Bi-Component Electrospinning

**Figure 16:**
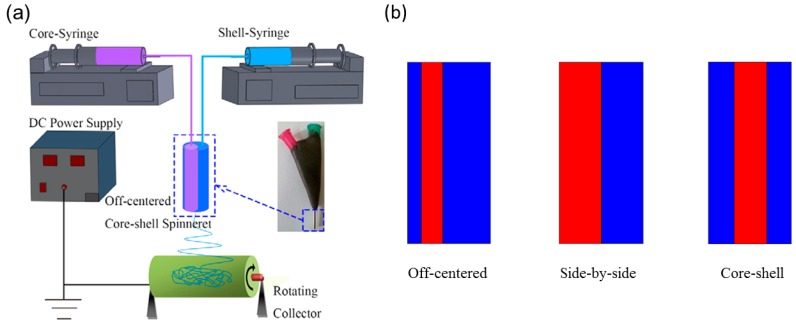
Schematic illustration of bi-component electrospinning setup and the internal structure of the electrospun fibers. (**a**) Schematic illustration of experimental setup of the bi-component electrospinning; (**b**) The schematic illustration of the off-centered, side-by-side and core-shell structure of the fiber. The blue and red represent different polymers. (**a**) is from [124], reprinted with permission, copyright (2015) American Chemical Society.

It is straightforward to extrude different polymer solutions together into one single jet to produce a heterogenous fiber, and the adjustable experiment setup of electrospinning makes it convenient to accomplish this. By using a multichannel springe where each polymer solution can flow separately in the spring and merge together at the outlet (Figure 16a), it is possible to acquire fibers with various internal structures such as core-shell [123], off-centered [124] and side-by-side [125,126] (Figure 16b). The constitution usually includes a soft elastomer and a rigid elastomer. The differential shrinkage between the two polymers during solution’s evaporation leads to the intrinsic curvature and coils the straight fiber accordingly. 

**Figure 17:**
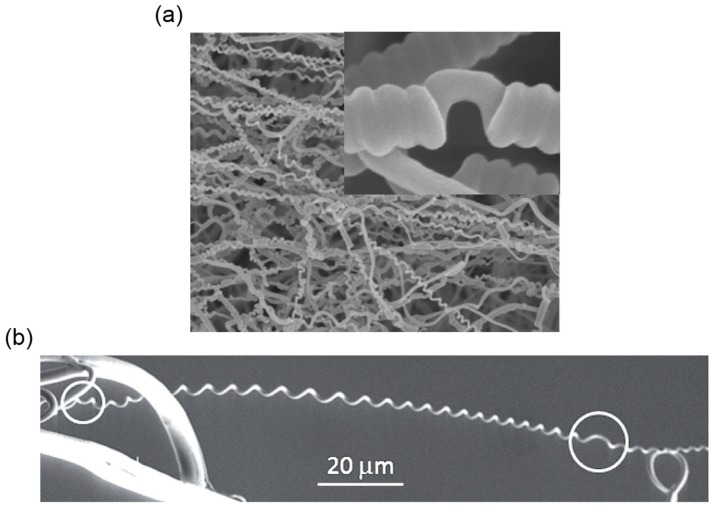
The electrospun fibers coil themselves with perversions. (**a**) Scanning electronic microscopy (SEM) image of TPU/Nomex nanofibers produced from side-by-side electrospinning; The inset shows a perversion inside the nanospring; (**b**) SEM image of the electrospun cellulose fibers, the perversion is underscored with white circles. (**a**) is from [127], reprinted with permission, copyright © 2009, John Wiley and Sons; (**b**) is from [128], reprinted with permission of Royal Society of Chemistry.

Chen et al. fabricated a hybrid fiber composed of a thermoplastic elastomer polyurethane (TPU) and a thermoplastic stiff polymer such as poly(*m*-phenylene isophthalamide) (Nomex), polylactide (PLA) and polysulfonamide (PSA) based on the bi-component electrospinning [123,127]. The synthesized fibers coil themselves and form perversions, resembling tendril’s coiling (Figure 17a). One would expect a side-by-side alignment will offer the largest intrinsic curvature compared to the off-centered and core-shell structures if other parameters are the same, and indeed in experiment the helix with the side-by-side alignment yields more coiling turns than the off-centered and the core-shell fibers. 

Lin et al. produced tightly coiled fibers with an average diameter 240 nm and a helix’s diameter 500 nm by co-electrospinning the elastomeric polyurethane (PU) and the thermoplastic polyacrylonitrile (PAN) based on a microfluidic device [125]. Chen et al. also fabricated nano-mat consisting of helical TPU/Nomex fibers from side-by-side electrospinning or straight TPU/Nomex fibers and performed tensile tests on both [127]. The results show that the coiled fibers with perversions have better mechanical properties than the straight fibers in terms of the elongation and toughness (Table 3), indicating that the synthesized coiled nanofibers have potential applications in protective fabrics, advanced filters and scaffold in tissue engineering [129].

##### Electrospinning Liquid Crystalline Cellulose

Other than the strategy of combining multiple polymers, one can use the cholesteric liquid crystalline cellulose solution to build a heterogenous structure once it is extruded from the nozzle [130]. This method relies on the cellulose’s cholesteric-to-nematic phase transition under shear deformation from the nozzle’s wall, during which the chiral molecular direction is untwisted and aligned parallel to the wall. The interaction between the molecular chirality of the cellulose and the shear alignment of the wall will create a helical, hard disclination line shifted from the fiber’s axis [131]. The disclination line is more rigid and undergoes less shrinkage compared to the rest of the fiber, providing an intrinsic curvature and a torque as well. 

Godinho et al. electrospun the acetoxypropylcellulose (APC) fibers and collected them in a such way that both ends of the fibers were bonded to the electrode collectors [128]. Decreasing the distance between two electrode collectors, the straight fibers will coil themselves into helices with perversions (Figure 17b). Besides, the morphological helicity is determined by the intrinsic curvature and torsion and irrelevant to the molecular chirality, demonstrated by the fact the fiber made of right-handed chiral molecules could either twist into a right-handed or left-handed helical shape [128].

##### Selective UV Irradiation

**Figure 18:**
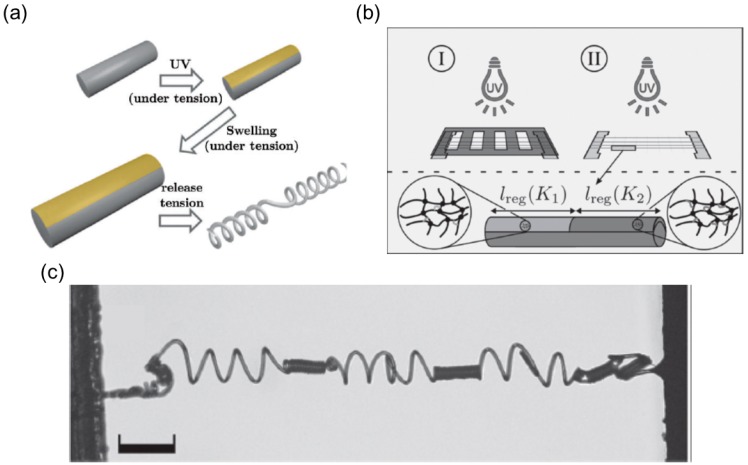
The electrospun fibers with further UV crosslinking. (**a**) Schematic illustration of the heterogenous structure induced by UV irradiation; (**b**) Schematic illustration of the two-step UV crosslinking in generating regions with different intrinsic curvatures; (**c**) Polarized light microscopy (POM) image of electrospun fibers separated by high-intrinsic-curvature regions and low-intrinsic-curvature regions. (**a**) is from [132], reprinted with permission, copyright © 2013, John Wiley and Sons; (**b**,**c**) are from [133], reprinted with permission, copyright © 2017, John Wiley and Sons.

A heterogeneous fiber can be produced from a homogeneous fiber if certain regions are selectively modified. The poly-(propylene oxide)-based triisocyanate-terminated prepolymer (PU)/hydroxyl-terminated polybutadiene (PBDO) fibers allow further crosslinking reaction under the UV irradiation due to the existing double bonds, and the regions exposed are more rigid than the protected part, eliciting the intrinsic curvature [132]. By exposing one side of the PU/PBDO fiber to UV irradiation, Trindade et al. obtained fibers with side-by-side internal structures in which the UV-crosslinked side was more rigid and stood less shrinkage [132]. The stretched fibers between two targets can coil into a helical spring with perversions as the distance between the fiber’s ends decreases (Figure 18a).

The advantage of this light-trigger crosslinking is that the intrinsic curvature’s distribution can be rationally designed with high spatial resolution along one single fiber by selectively exposing certain regions to UV light, using photomasks cut by high-precision machine. Silva et al. used the two-step light exposure to generate fibers with nonuniform distribution of intrinsic curvature [133]. In the first step, some areas from one side are protected from UV irradiation by photomasks while no masks are used in the second step, delivering all the exposed area equal irradiation. As a result, the areas exposed to light in the first step become more rigid than the rest, bringing out separative sections with high and low intrinsic curvatures (Figure 18b). Since the critical pulling force of this bending instability *T* is quadratically related to the intrinsic curvature *K* ($T\sim K^{2}$) [15,133], the regions with high intrinsic curvature will coil first when the fiber gets released before the regions with low intrinsic curvature, and the coiling will be more severe in high-intrinsic-curvature regions than the low-intrinsic-curvature regions (Figure 18c). 

**Figure 19:**
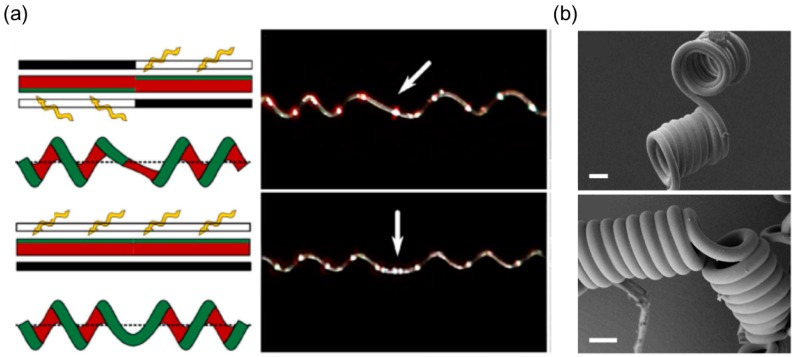
The asymmetry and symmetry perversion in electrospun fibers under UV irradiation. (**a**) Left: schematic illustration of asymmetry perversion (top) and symmetry perversion (bottom) guided by the UV irradiation, right: experimental figures of asymmetry (top) and symmetry perversion (bottom). The perversion is pointed out by white arrows; (**b**) SEM images of the asymmetry (top) and symmetry perversion (bottom) in electrospun fibers. The scale bar is 10 μm. (**a**,**b**) are from [134], reprinted with permission from Nature Publishing Group.

This light patterning methodology also offers an answer to a fundament question: whether the ‘perversion’ shape observed in tendrils is the only possibility to smoothly link two helices with opposite handedness? Theoretically, there exist a range of different configurations for a perversion and the natural perversion is the case with mirror symmetry with respect to the axial plane at the perversion’s center. For the asymmetry perversion, the normal direction of the curve switches direction at the linking point of the two helices, and such discontinuity can be realized by varying the direction of intrinsic curvature abruptly at the linking point. For an experimental verification, Silva et al. exposed the opposite sides of two neighboring sections to UV light, and these two sections will intrinsically bend into opposite directions [134] (Figure 19a). After releasing the stretched fibers, an asymmetry perversion appears, and the axis of the connecting helices has a tilting angle. Unlike the symmetry perversion that rotates around the helix’s axis during the pulling, the asymmetry perversion keeps asymmetry when the helix is stretched: the location of the perversion’s center keeps unchanged while the sections to its right and left rotate in counterphase. When the fiber is absent from stretching, the asymmetry perversion shows a straight shape while the symmetry perversion bends itself (Figure 19b).

## 4. Summary and Outlook

Nature develops fascinating biological mechanisms to form complex morphologies, inspiring innovative fabrication of sophisticated structures in artificial systems. Among numerous biomimetic cases, the synthesis of helical structures mimicking the chiral opening process of *Bauhinia variegata*’s pod and the coiling of plant’s tendril has recently proven to be a great success. The morphogenesis of these plants provides a generic principle to produce a helical structure—an initially flat, ribbon-like or straight, rod-like structure will transform its shape into a helix driven by the intrinsic configuration. This morphing mechanism has several advantages. First, the precursor can be a flat ribbon or a straight rod, compatible with various developed techniques such as layer-by-layer deposition and electrospinning. Second, like the differential growth in plants, the differential response of the stimuli-responsive materials such as hydrogels and liquid crystal elastomers can be exploited to precisely construct an intrinsic shape guided by the formulated theories [11,23,57]. In addition, by controlling the external stimuli, these artificial helices can wind or unwind themselves to output actuations including contraction, elongation and rotation. Third, this shape transformation is not restricted by the shape’s dimension, broadening its accessibility across different length scales. Fourth, the geometric parameters of the target helix can be tuned on demand by varying the precursor’s dimension or the intrinsic configuration, providing feasible pathways to programmable fabrication of the helical structures. Given these excellent features, it is promising that these laboratory-scale works will herald the future applications of biomimetic helical structures in various technology fields, such as the propulsion tail in the micro-robot that can swim in human body for biomedical purpose [135,136].

These biomimetic examples enlightened by *Bauhinia variegata* and tendrils can also exhibit unexpected yet interesting properties compared to their biological counterpart, broadening the design space for future applications. For example, multiple perversions tightly located to each other along one single strip can be produced in the bilayer elastomer strip when the stretch ratio χ is large and the aspect ratio of cross section h/w is small, whereas perversions observed in tendrils are always sparsely positioned. In addition, asymmetry perversion can be achieved by abruptly changing the bending direction of the intrinsic curvature at some point, owing to the UV crosslinking with high spatial resolution. However, this morphology is not expected to be observed in tendrils since such a discontinuity is absent from nature.

Although significant advances have been made in the synthesized helical structures inspired by these plants, some unique behaviors originating from the shape-shifting mechanisms have still not been fully exploited for better functionality, such as bistability [11,25,45,137]. For the helix driven by the intrinsic saddle shape, two stable solutions emerge when the dimensionless width exceeds the threshold, and two stable helices can exist under the same condition. The switch between these two stable states, the so-called snap-through, is of great benefit in terms of achieving fast and large actuation without continuing energy input. For instance, a bistable beam clamped at both ends can either bend upwards or downwards when two ends are brought to each other [138]. It is found that it takes less than 0.02 s for one of the curved shapes to switch to the other and the largest displacement during this process is around 10 mm, yielding an average actuation speed of 0.5 m/s [139]. This switch is faster than any of the shape transformations driven by external stimuli reported in this article, however, to the authors’ knowledge it has not been extensively used to realize fast snaps of helical structures. The harnessing of these distinguished properties will likely provide a much enriched design space for smart structures and devices with a wide range of applications.

## Figures and Tables

**Table 1:** Helical structures mimicking *Bauhinia variegata* based on hydrogels, liquid crystal networks/elastomers, and shape memory polymers.

	Stimulus	Isotropic/Anisotropic	Approximate Size	Actuation Time	Reversible
**Hydrogels**	humidity	isotropic: [26,27]	~45mm [26,27]	>7 min [26] ~47 min [27]	yes
	thermal	isotropic: [28,29]	several hundred micron (<1 mm) [29]	2 min [29]	
	pH	isotropic [30]	20–70 mm [30]	1–30 min [30]	
**LCNs/LCEs**	thermal	anisotropic: [31,32,33,34,35,36] Mix [37]	5–25 mm [31,32,33,34,35,36,37]	unknown	yes [31,32,34,35,36] depending on cooling speed [33]
	UV light	anisotropic [38,39,40,41,42]	8–40 mm [38,39]	a few mins [38] 12 mins [39]	yes
	chemical		10 mm [40]	4–10 s [40]	
	humidity		20 mm [41]	unknown	
	water/acetone		20–30 mm [42]	=< 10 s [42]	
**SMPs**	water	anisotropic [43,44]	10–20 mm [43]	unknown [43]	no
	thermal		5–30 mm [44]	unknown [44]	

**Table 2:** Performance of a hierarchical CNT fiber (composed of 20 primary fibers) and a primary CNT fiber (composed of 20 CNT sheets) when exposed to dichloromethane [113].

	Maximum Contractive Strain	Maximum Contractive Stress	Maximum Contractive Strain Rate	Maximum Contractive Stress Rate	Maximum Rotatory Speed	Reversibility
**Secondary Fiber**	15%	1.5 Mpa	330%/s	8.0 MPa/s	6361rpm	>30 cycles
**Primary Fiber**	9%	<1.0 MPa	30%/s	2.5 MPa/s	760 rpm	<15 cycles

**Table 3:** Mechanical properties of nanomats composed of helical TPU/Nomex fibers (side-by-side) or straight TPU/Nomex fibers [127].

	Tensile Strength (MPa)	Elongation (%)	Toughness (MPa)	Maximum Storage Modulus (GPa)
**Helical Fiber**	151	97	102	6.70
**Straight Fiber**	202	33	59.0	5.15
